# Transient Receptor Potential Ankyrin 1 (TRPA1)—An Inflammation-Induced Factor in Human HaCaT Keratinocytes

**DOI:** 10.3390/ijms22073322

**Published:** 2021-03-24

**Authors:** Samu Luostarinen, Mari Hämäläinen, Eeva Moilanen

**Affiliations:** The Immunopharmacology Research Group, Faculty of Medicine and Health Technology, Tampere University and Tampere University Hospital, 33014 Tampere, Finland; samu.luostarinen@tuni.fi (S.L.); mari.hamalainen@tuni.fi (M.H.)

**Keywords:** transient receptor potential ankyrin 1 (TRPA1) cation channel, inflammation, tumor necrosis factor (TNF), calcineurin inhibitors, glucocorticoids

## Abstract

Transient receptor potential ankyrin 1 (TRPA1) is an ion channel mainly studied in sensory neurons where it mediates itch, pain and neurogenic inflammation. Recently, some nonneuronal cells have also been shown to express *TRPA1* to support inflammatory responses. To address the role of TRPA1 in skin inflammation, we aimed to investigate *TRPA1* expression in keratinocytes. HaCaT cells (a model of human keratinocytes) and skin biopses from wild-type and *TRPA1* deficient mice were used in the studies. *TRPA1* expression in nonstimulated keratinocytes was very low but significantly inducible by the proinflammatory cytokine tumor necrosis factor (TNF) in an nuclear factor kappa B (NF-κB), and mitogen-activated protein (MAP) kinase (p38 and c-Jun N-terminal kinase, JNK)-dependent manner. Interestingly, drugs widely used to treat skin inflammation, the calcineurin inhibitors tacrolimus and cyclosporine and the glucocorticoid dexamethasone, significantly decreased *TRPA1* expression. Furthermore, pharmacological inhibition and genetic deletion of TRPA1 reduced the synthesis of TNF-induced monocyte chemoattractant protein 1 (MCP-1) in keratinocytes and mouse skin biopsies. In conclusion, these findings point to an inflammatory role for TRPA1 in keratinocytes and present TRPA1 as a potential drug target in inflammatory skin diseases.

## 1. Introduction

Transient Receptor Potential Ankyrin 1 (TRPA1) is an ion channel localized to the plasma membrane. It mediates ion currents, mainly Ca^2+^ and Na^+^, but also other cations such as Zn^2+^. [1] Previously, the majority of research has focused on TRPA1 in sensory neurons. Indeed, *TRPA1* expression is well characterized in Aδ and C-type fibers, where it functions mediating pain and hyperalgesia [2,3]. The main physiological function of TRPA1 is believed to be a neuronal chemosensor, sensing and responding to noxious compounds [1]. In addition, TRPA1 has been shown to participate in the generation of neurogenic inflammation in sensory neurons where elevation of intracellular calcium levels through TRPA1 activation promotes exocytosis of proinflammatory neuropeptides [4]. TRPA1 expression has been characterized also in some nonneuronal cells such as synoviocytes [5] and chondrocytes [6]. In these cells, TRPA1 has been shown to promote inflammatory responses [5,6] but its physiological functions in nonneuronal cells remain unclear.

TRPA1 is activated by various stimuli, including chemical, mechanical and thermal irritation [7]. Of these functions, the chemosensory function of TRPA1 is best characterized. Indeed, the list of known TRPA1 ligands is rather long, including exogenous and endogenous compounds. The exogenous activators include substances such as allyl isothiocyanate (AITC) [8] from mustard oil and allicin [9] from garlic. Interestingly, the endogenous TRPA1 activator compounds include inflammation associated factors, particularly reactive oxygen (ROS) and nitrogen (RNS) species and their derivatives [7,10,11,12,13]. In addition, TRPA1 modulation extends to indirect sensitization via G protein-coupled receptor (GPCR) pathways. For example, the inflammatory mediators bradykinin and prostaglandin E_2_, and the proteases trypsin and tryptase, via their respective GPCRs, sensitize TRPA1 through activating the intracellular protein kinase A (PKA) and phospholipase C (PLC) signaling pathways [14,15,16]. PKA sensitizes TRPA1 through direct phosphorylation of certain TRPA1 amino acid residues [17], while PLC seems to mainly act via depleting phosphatidylinositol 4,5-bisphosphate (PIP2), which is known to inhibit TRPA1 [15].

TRPA1 activation by exogenous or endogenous agonists has been reported to induce and/or support inflammation in various models. Topical application of AITC is known to induce inflammation in a TRPA1-dependent manner as judged by pharmacological and genetic evidence [3,18]. TRPA1-mediated inflammation has also been observed in carrageenan-induced paw edema [19] and urate crystal [20,21] and monosodium-iodoacetate (MIA)-induced arthritis [22]. TRPA1 may also be involved in some inflammatory skin disorders, as neurogenic inflammation is known to play a role in cutaneous inflammation [4]. In addition, TRPA1 has been reported to promote the inflammatory response and itch in models of allergic contact dermatitis, atopic dermatitis and cytokine-induced itch [23,24,25,26,27], while a protective role has been suggested for TRPA1 in the imiquimod-induced model of psoriasiform dermatitis [28].

Although *TRPA1* expression in human keratinocytes has been reported [29,30], not much is known about the underlying mechanisms. As TRPA1 seems to have a significant role in models of inflammatory skin conditions such as atopic dermatitis and allergic contact dermatitis, and keratinocytes contribute to the pathogenesis of these skin conditions, we aimed to study *TRPA1* expression in human HaCaT keratinocytes exposed to inflammatory stimuli.

## 2. Results

### 2.1. TRPA1 Expression Is Enhanced by TNF in Human HaCaT Keratinocytes

**Figure 1:**
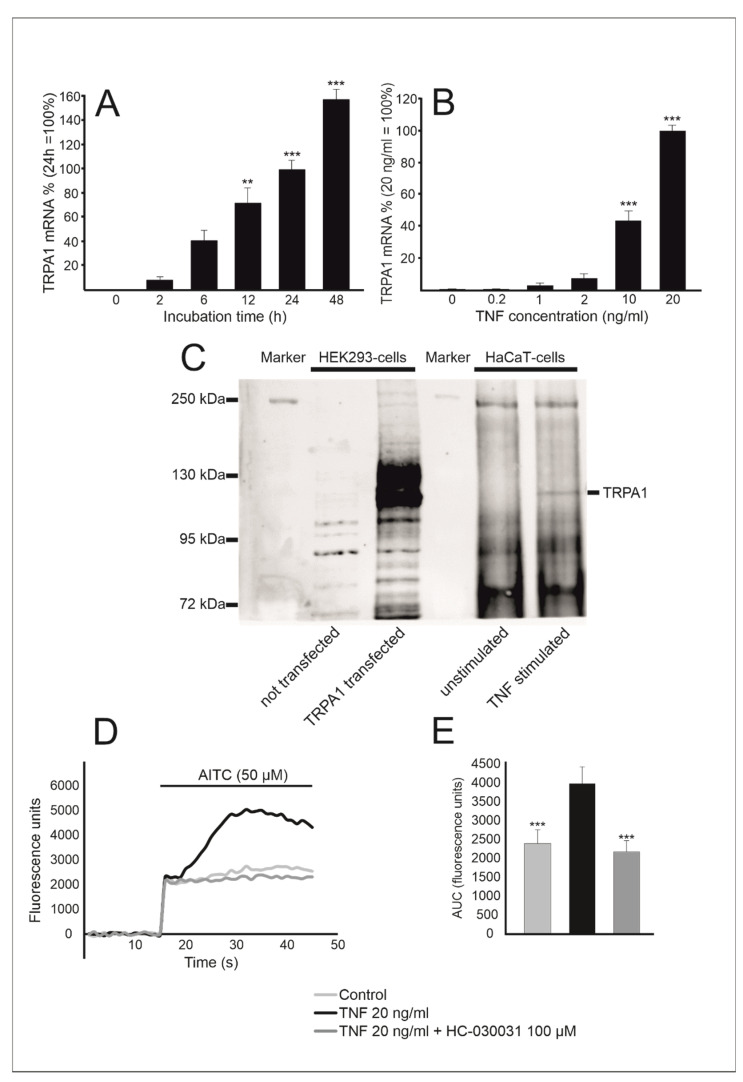
Tumor necrosis factor (TNF) induces transient receptor potential ankyrin 1 (*TRPA1*) expression in human HaCaT keratinocytes in a time and dose-dependent manner. HaCaT cells were stimulated with TNF (20 ng/mL) for indicated times (**A**) or with an increasing concentration of TNF (from 0.2 ng/mL to 20 ng/mL) for 24 h (**B**). Thereafter total RNA was extracted and *TRPA1* expression was measured by RT-PCR. Results were normalized against *GAPDH* mRNA. *TRPA1* mRNA level at 24 h (**A**) and with 20 ng/mL (**B**) was set as 100% and the other values are given in relation to that value. Results are expressed as mean + SEM, *n* = 4. Statistical significance was calculated against 2 h (**A**) or control without added TNF (**B**) using one-way ANOVA with Bonferroni’s post-test. ** = *p* < 0.01, *** = *p* < 0.001. In (**C**), HaCaT keratinocytes were cultured with TNF (20 ng/mL) for 48 h. After protein extraction and immunoprecipitation, TRPA1 protein (indicated in image) was detected using Western blotting. HEK293 cells transfected with human *TRPA1* plasmid were used as a positive control. The blot is a representative of three distinct experiments with similar results. In (**D**,**E**) is shown that TNF-treated HaCaT-cells exhibited a robust intracellular Ca^2+^ increase in response to allyl isothiocyanate (AITC) stimulation, effectively inhibited by HC-030031, suggesting that the upregulated TRPA1 is functional. Intracellular Ca^2+^ levels were measured utilizing the Ca^2+^ indicator Fluo 3-AM. HaCaT keratinocytes were cultured with or without TNF (20 ng/mL) for 24 h. Thereafter, the cells were incubated in the presence or absence of the TRPA1 antagonist HC-030031 (100 µM) for 30 min before the TRPA1 agonist AITC (50 µM) was applied and measurements were continued for 30 s. In (**D**) is shown a representative measurement, and in (**E**) area under curve analysis of the experiment in (**D**) is presented. Results in (**E**) are expressed as mean + SEM, *n* = 8. Statistical significance was calculated against cells cultured with TNF and measured in the absence of HC-030031 using one-way ANOVA with Bonferroni’s post-test, *** = *p* < 0.001.

In basal conditions, *TRPA1* expression in HaCaT keratinocytes was barely measurable by RT-PCR. When tumor necrosis factor (TNF) was added into the culture, *TRPA1* expression was enhanced in a dose and time-dependent manner (Figure 1). The effect of other inflammatory factors, namely IL-1β (100 pg/mL), LPS 10 (ng/mL), resistin (1 µg/mL), leptin (10 µg/mL), IL-6 (100 ng/mL), IL-13 (10 ng/mL) and IFN-γ (10 ng/mL), on *TRPA1* expression was also studied, but statistically significant upregulation of *TRPA1* expression was not observed.

To confirm *TRPA1* expression at the protein level, we carried out Western blot analyses using HEK-293 cells transfected with the human *TRPA1* plasmid as a positive control. TRPA1 protein expression was upregulated in HaCaT cells stimulated with TNF but not detectable in unstimulated cells. (Figure 1C).

In addition, the functionality of the TRPA1 ion channel was investigated using the Fluo 3-AM intracellular Ca^2+^ measurement. HaCaT cells were cultured with or without TNF (20 ng/mL) for 24 h. Thereafter, the cells were preincubated with or without the TRPA1 blocker HC-030031 (100 µM) for 30 min before the TRPA1 agonist AITC (50 µM) was applied. We detected a significant increase in intracellular Ca^2+^ concentration upon AITC application in cells cultured with TNF, which was completely reversible by HC-030031 treatment, indicating that TNF increases the expression of functional TRPA1 channel (Figure 1D,E).

### 2.2. TRPA1 Expression Is Upregulated by NF-κB and MAP Kinase Pathways

**Figure 2:**
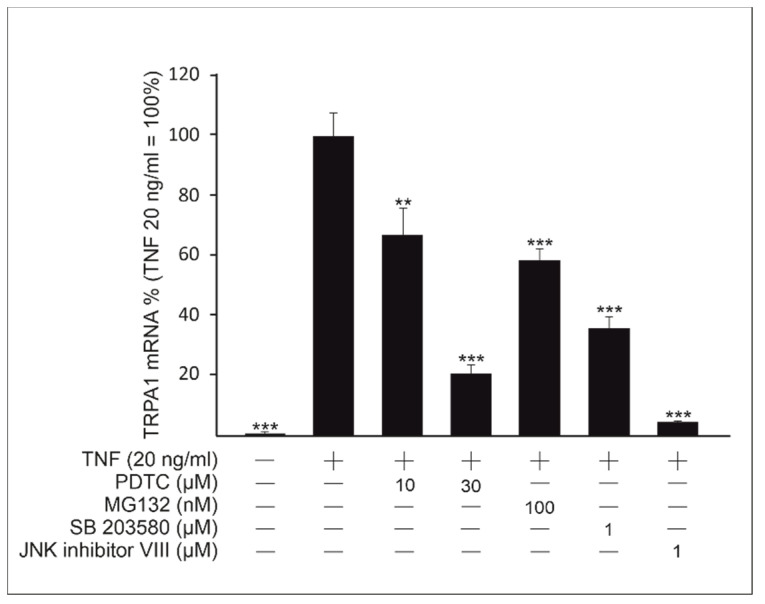
Transient receptor potential ankyrin 1 (*TRPA1*) upregulation by tumor necrosis factor (TNF) is mediated by nuclear factor kappa B (NF-κB), and p38 and c-Jun N-terminal kinase (JNK) mitogen-activated protein kinase (MAPK) pathways. *TRPA1* expression was induced by TNF (20 ng/mL), and pharmacological inhibitors of the signaling routes were applied. p38 MAPK inhibitor SB 203580 (1 µM), JNK inhibitor VIII (1 µM) and NF-κB inhibitors MG132 (100 nM) and pyrrolidinedithiocarbamate ammonium (PDTC; 10 and 30 µM) were used. Incubations were continued for 24 h, after which total RNA was isolated and *TRPA1* expression was measured using RT-PCR. Results were normalized against *GAPDH* mRNA. TNF-induced *TRPA1* expression in the absence of the signaling inhibitors was set as 100% and the other values are given in relation to that value. Results are expressed as mean + SEM, *n* = 4. Statistical significance was calculated against cells stimulated with TNF only using one-way ANOVA with Bonferroni’s post-test. ** = *p* < 0.01, *** = *p* < 0.001.

After observing the increased expression of *TRPA1* in response to TNF, we continued by studying which intracellular signaling pathways relevant to inflammation might contribute to *TRPA1* upregulation under TNF stimulation. The pharmacological inhibitors MG132 and pyrrolidinedithiocarbamate ammonium (PDTC), c-Jun N-terminal kinase (JNK) inhibitor VIII and SB 203580 were used to study the role of nuclear factor kappa B (NF-κB), and JNK and p38 MAP kinases, respectively. Both NF-κB inhibitors MG132 and PDTC inhibited TNF-induced *TRPA1* expression. In addition, JNK inhibitor VIII and SB 203580 had a statistically significant inhibitory effect on TNF-induced *TRPA1* expression, indicating a role for NF-κB as well as JNK and p38 MAP kinases in upregulating TRPA1 in these cells (Figure 2).

### 2.3. The Immunosuppressive Drugs Dexamethasone, Cyclosporine and Tacrolimus Inhibit TNF-Induced TRPA1 Expression

**Figure 3:**
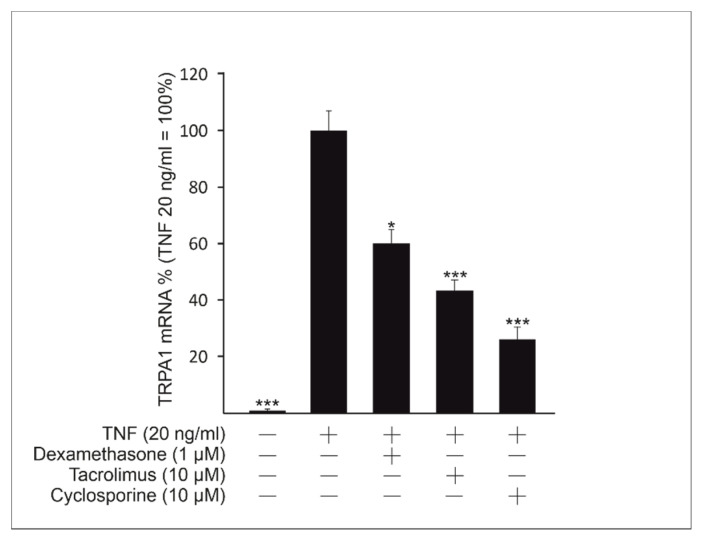
Dexamethasone and calcineurin/nuclear factor of activated T-cells (NFAT) inhibitors cyclosporine and tacrolimus inhibit transient receptor potential ankyrin 1 (*TRPA1*) expression under tumor necrosis factor (TNF) stimulation. HaCaT cells were cultured with TNF in the absence or in the presence of the drugs tested for 24 h. Thereafter total mRNA was extracted and *TRPA1* expression was measured using RT-PCR. Results were normalized against *GAPDH* mRNA. TNF-induced *TRPA1* expression in the absence of the tested drugs was set as 100% and the other values are given in relation to that value. Results are expressed as mean + SEM, *n* = 4. Statistical significance was calculated against cells stimulated with TNF only using one-way ANOVA with Bonferroni’s post-test. * = *p* < 0.05, *** = *p* < 0.001.

As *TRPA1* was shown to be induced by the key inflammatory cytokine TNF, and its expression was mediated via inflammatory signaling pathways, we aimed to investigate whether immunosuppressive drugs affect *TRPA1* expression. Effects of the calcineurin/nuclear factor of activated T-cells (NFAT) pathway inhibitors cyclosporine and tacrolimus, and the potent glucocorticoid dexamethasone, drugs widely used in the treatment of inflammatory skin conditions, were studied. Interestingly, we found that both cyclosporine and tacrolimus significantly inhibited TNF-induced *TRPA1* upregulation (Figure 3). Dexamethasone was also found to inhibit *TRPA1* expression (Figure 3).

### 2.4. TRPA1 Mediates the Production of the Chemokine MCP-1 in TNF-Stimulated HaCaT Cells and in Mouse Skin Ex Vivo

**Figure 4:**
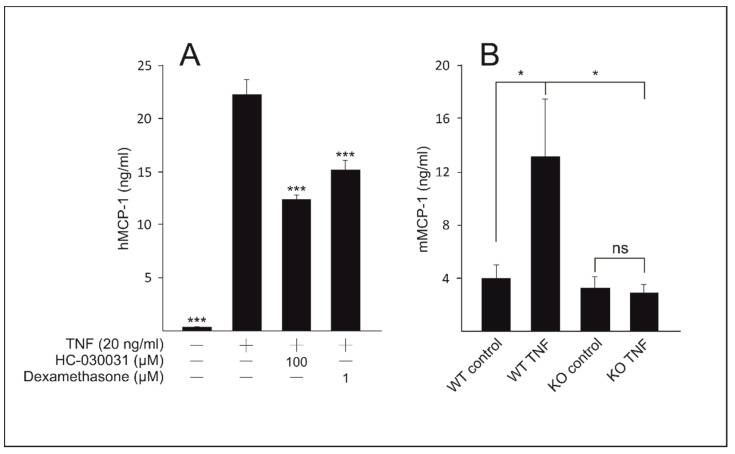
Transient receptor potential ankyrin 1 (TRPA1) mediates tumor necrosis factor (TNF)-induced production of monocyte chemoattractant protein 1 (MCP-1). (**A**) HaCaT cells were treated with the TRPA1 antagonist HC-030031 (100 µM) or the glucocorticoid dexamethasone (1 µM) and stimulated with TNF (20 ng/mL) for 24 h. (**B**) Skin samples from wild type (WT) and *TRPA1* deficient (knockout, KO) mice were stimulated with TNF (20 ng/mL) for 21 h. MCP-1 concentrations in the culture medium samples were measured by enzyme-linked immunosorbent assay (ELISA). Results are expressed as mean + SEM, *n* = 4 (**A**) and *n* = 4–6 (**B**). Statistical significance was calculated against samples stimulated with TNF only using one-way ANOVA with Bonferroni’s post-test (**A**) or in **(B)** using repeated measures two-way ANOVA with Bonferroni’s post-test. * = *p* < 0.05, *** = *p* < 0.001.

To preliminarily assess the effects of TRPA1 in the skin, we measured the production of monocyte chemoattractant protein 1 (MCP-1) in the HaCaT keratinocyte culture and in ex vivo mouse skin sample supernatants. Interestingly, significantly decreased MCP-1 levels were found in the cell cultures under treatment with the TRPA1 antagonist HC-030031 as compared to stimulation with TNF only. The result was reproduced in mouse skin samples ex vivo, where TNF was found to enhance MCP-1 production in samples from wild type (WT) but not in those from *TRPA1* deficient (knockout, KO) mice (Figure 4). 

## 3. Discussion

The current findings reveal TRPA1 as a potential factor in skin inflammation. *TRPA1* expression was barely detectable in basal conditions in human HaCaT keratinocytes but was highly induced by TNF. Further, we found NF-κB, and p38 and JNK MAP kinase pathways to mediate TNF-induced *TRPA1* upregulation as judged by pharmacological evidence. Intriguingly, the anti-inflammatory drugs cyclosporine, tacrolimus and dexamethasone (which are all used to treat inflammatory skin diseases) significantly inhibited TNF-induced *TRPA1* upregulation. Finally, we found a role for TRPA1 in mediating MCP-1 production in human keratinocytes and ex vivo skin samples of mice, as assessed by immunoassay.

After being first found in human fibroblasts [31], TRPA1 has mainly been investigated in sensory neurons where it is expressed in Aδ and C-fibers [1,32,33]. More recently, TRPA1 has been studied also in non-neuronal cells such as chondrocytes [6] and synoviocytes [5]. A study by Atoyan et al. [30] first implicated *TRPA1* expression in human skin. Thereafter, *TRPA1* expression in the skin was reported in mast cells [25], melanocytes [34] and keratinocytes [29]. In nonneuronal cells, TRPA1 has been shown to support proinflammatory responses [25,29,35]. In addition, TRPA1 has been found to contribute to many preclinical models of inflammatory diseases such as allergic contact dermatitis [24], ovalbumin-induced allergic inflammation [36], carrageenan-induced acute inflammation [19], gout [20,21], osteoarthritis [22] and inflammatory bowel disease [37]. In the present study, we found that *TRPA1* is, under basal conditions, barely expressed but strongly inducible by the cytokine TNF in a time and dose-dependent manner in human HaCaT keratinocytes. These results are supported by the previous observations made by Hatano et al. [5] that TNF induces *TRPA1* in human synoviocytes. Of interest is that TNF-blockers are successfully used in the treatment of inflammatory skin diseases such as psoriasis [38]. Our finding that TNF significantly enhances *TRPA1* expression supports the hypothesis that TRPA1 is an inflammatory factor induced upon inflammation.

Having established *TRPA1* expression in human keratinocytes and its upregulation by TNF, we explored the mechanisms of TNF-induced *TRPA1* expression in these cells. Indeed, the previous knowledge on intracellular mechanisms mediating *TRPA1* upregulation has been scarce and, in keratinocytes, nonexistent. NF-κB is a crucial inflammatory transcription factor induced under inflammatory conditions regulating a number of genes [39]. In the present study, we found that the NF-κB inhibitors PDTC and MG132 significantly inhibited TNF-induced *TRPA1* expression in human keratinocytes. These results are supported by observations that the *TRPA1* gene promoter includes NF-κB binding sites, and TNF-induced *TRPA1* upregulation is partly mediated via NF-κB in human synoviocytes [5]. We also found that TNF-induced *TRPA1* upregulation is at least partly regulated by the MAP kinase pathways p38 and JNK, as assessed by experiments utilizing their pharmacological inhibitors SB 203580 and JNK inhibitor VIII, respectively. Both pathways are relevant in inflammation, and their inhibition holds potential for treatment of inflammatory disorders [40]. These findings together suggest that TRPA1 is an inflammation-inducible factor in human HaCaT keratinocytes.

Calcineurin inhibitors, including cyclosporine and tacrolimus, are a class of immunosuppressive and anti-inflammatory drugs used in the treatment of atopic dermatitis and psoriasis along with their original indication to prevent transplant rejection. Traditionally calcineurin inhibitors were thought to act mainly on T-helper cells and suppress the production of IL-2 and other inflammatory cytokines. [41] More recently, the function of the calcineurin/NFAT pathway has also been established in human keratinocytes, regulating cell growth and differentiation, and cytokine expression, possibly accounting for some of the beneficial effects of these drugs in skin diseases [42,43,44]. Interestingly, we found here that both cyclosporine and tacrolimus inhibit the inflammatory upregulation of *TRPA1* in human keratinocytes, which is a novel finding and may contribute to their pharmacological effects in skin diseases. 

Glucocorticoids are topically used widely in inflammatory skin diseases [45]. We observed a significant inhibition of the inflammatory *TRPA1* upregulation by the potent glucocorticoid dexamethasone. This is in agreement with our recent findings in chondrocytes [46]. Based on the current discoveries, two different groups of anti-inflammatory compounds, namely glucocorticoids and calcineurin inhibitors, seem to downregulate *TRPA1* expression in keratinocytes, potentially accounting for some of their anti-inflammatory effects.

TRPA1 activation leads to cellular influx of cations, especially Ca^2+^ and Na^+^. Calcium widely regulates gene expression [47] and, accordingly, TRPA1 activation has been reported to regulate the expression of certain inflammatory factors [6,20,25,29,30]. Here we showed that TNF-stimulated human keratinocytes produce decreased amounts of MCP-1 under TRPA1 antagonist (HC-030031) therapy, indicating that TRPA1 supports the production of MCP-1 upon TNF stimulation in these cells. Accordingly, we found that in skin samples from mice, TNF-induced MCP-1 production was significantly lower in *TRPA1* KO mice compared to WT mice. MCP-1, also known as chemokine (C-C motif) ligand 2 (CCL2), is a potent chemokine linked to many inflammatory conditions mainly attracting monocytes/macrophages to the inflammatory tissue [48]. Intriguingly, MCP-1 is seen as a major factor also in psoriasis, where keratinocytes are an important source of MCP-1, contributing to lesion formation [49]. Our results are supported by previous results showing that HC-030031 attenuated MCP-1 production in response to urate crystals [20]. Thus, we suggest that TRPA1 supports the production of MCP-1 in keratinocytes in inflammatory conditions, potentially linking TRPA1 to the pathogenesis of psoriasis and other inflammatory conditions of the skin.

Taken together, these results suggest that in homeostatic conditions *TRPA1* is barely expressed in human keratinocytes but is induced upon inflammatory conditions via TNF and key inflammatory signaling pathways, while *TRPA1* expression is downregulated by calcineurin/NFAT inhibitors and the glucocorticoid dexamethasone. Once induced, TRPA1 supports the production of MCP-1. Intriguingly, the present study highlights multiple mechanisms affecting *TRPA1* expression that are also modulated by immunosuppressive and anti-inflammatory drugs used in the clinic. Interestingly, TNF-inhibitors have shown efficacy and are successfully used in psoriasis and other inflammatory skin conditions [38]. Calcineurin/NFAT inhibitors are indicated in atopic dermatitis and some other skin diseases [41]. The use of topical glucocorticoids is widespread and effective in inflammatory skin conditions [45]. In addition to our data that *TRPA1* is an inflammation-inducible gene and supports inflammatory responses, TRPA1 has been shown to play a role in murine models of allergic contact dermatitis [23,24] and atopic dermatitis [25]. Thus, these findings together point to an inflammatory role for TRPA1 in keratinocytes and present TRPA1 as a potential drug target in inflammatory skin diseases.

## 4. Materials and Methods

### 4.1. Cell Culture

HaCaT cells (Cell Lines Service, Eppelheim, Germany) were cultured according to Wilson et al. [50] in low calcium conditions. During the experiments, the HaCat keratinocytes were treated with tumor necrosis factor-alpha (TNF) (R&D Systems Europe Ltd., Abingdon, UK), PDTC (Sigma-Aldrich, St. Louis, MO, USA), MG 132 (Tocris Bioscience, Bristol, UK), JNK inhibitor VIII (Calbiochem, San Diego, CA, USA), SB 203580 (Tocris Bioscience, Bristol, UK), dexamethasone (Orion corp., Espoo, Finland), tacrolimus (Calbiochem, San Diego, CA, USA), cyclosporine (Calbiochem, San Diego, CA, USA), HC-030031 (Sigma-Aldrich, St. Louis, MO, USA), or with their combinations as indicated.

Human embryonic kidney cells (HEK-293, American Type Culture Collection, Manassas, VA, USA) were cultured and transfected with human TRPA1 plasmid DNA (pCMV6-XL4 from Origene, Rockville, MD, USA) as described previously [6]. In brief, the cells were seeded in 6-well plates (1.2 × 10^6^ cells/well) 24 h prior to transfection. Cells were transfected for 16 h with 6 µg/well of human TRPA1 plasmid DNA using Lipofectamine 2000 (10 µL/mL; Invitrogen/Life Technologies, Carlsbad, CA, USA).

### 4.2. Animals

Wild type (WT) and *TRPA1* deficient (knockout, KO) female B6; 129P-Trpa1(tm1-Kykw)/J mice (Charles River Laboratories, Sulzfeld, Germany) were used in mouse skin sample experiments. Mice were housed under standard conditions (12–12 h light-dark cycle, temperature 22 ± 1 °C, and humidity 50–60%) with food and water provided ad libitum. The experiments were conducted in compliance with legislation for the protection of animals used for scientific purposes (Directive 2010/63/EU) and under Tampere University license EKS-2018 (issued 12 April 2018).

### 4.3. Mouse Skin Sample Culture

After euthanization, the back skin of the mice was shaved and a full thickness skin sample (diameter of 6 mm) for each experimental condition was removed from the back skin and cultured in Dulbecco’s Modified Eagle Medium (DMEM, Lonza, Bazel, Switzerland) containing 0.03 mM Ca^2+^, 4 mM L-glutamine, 250 ng/mL amphotericin B, 10% heat-inactivated fetal bovine serum, 100 U/mL penicillin and 100 mg/mL streptomycin (all from Gibco, Waltham, MA, USA and Invitrogen, Carlsbad, CA, USA). The skin samples were incubated with or without TNF (20 ng/mL) for 21 h and the culture media were collected for further measurements.

### 4.4. Western Blot Measurements

At the end of the experiments, samples were collected and TRPA1 immunoprecipitation and Western blot measurements were carried out as described previously [6]. In brief, HaCaT keratinocytes were cultured on 6-well plates. After 48 h of treatment with or without TNF (20 ng/mL), total protein was extracted. The extract from six wells was pooled to form one sample, and equal amount of each sample was subjected to TRPA1 immunoprecipitation. TRPA1 antibody SAB2105082 (2 µg/sample; Sigma-Aldrich) was added and the samples were incubated for 1 h at 4 °C in a rotator and thereafter Protein A/G PLUS-Agarose (sc-2003, Santa Cruz Biotechnology) was used to separate the immunocomplexes according to the manufacturer’s instructions. Afterwards, Western blot analysis was carried out as described previously [6] using the TRPA1 antibody NB110-40763 (Novus Biologicals, LCC, Littleton, CO, USA) and the goat antirabbit HRP-conjugate (sc-2004, Santa Cruz Biotechnology) as the primary and secondary antibody, respectively.

### 4.5. Fluo 3-AM Measurements

Intracellular Ca^2+^ measurements were carried out using Fluo 3-AM as described previously [18]. HaCaT cells were first loaded for 30 min with 4 µM fluo-3-acetoxymethyl ester (Fluo 3-AM, Millipore Sigma) and 0.08% Pluronic F-127^®^ in Hanks’ balanced salt solution (HBSS, Lonza, Verviers, Belgium) containing 1 mg/mL bovine serum albumin, 2.5 mM probenecid and 25 mM HEPES pH 7.2 (all from Millipore Sigma). Thereafter, free intracellular Ca^2+^ levels were analyzed using Victor3 1420 multilabel counter (Perkin Elmer, Waltham, MA, USA) at 485/535 nm (for excitation/emission, respectively). HaCaT cells were preincubated with HC-030031 (100 µM) or vehicle for 30 min before the TRPA1 agonist allyl isothiocyanate (AITC; 50 µM, Sigma-Aldrich) was added. The measurements were continued for 30 s.

### 4.6. Immunoassay

MCP-1 concentrations in the cell and tissue culture media were determined by enzyme-linked immunosorbent assay (ELISA) with reagents purchased from R&D Systems Europe Ltd., Abingdon, UK.

### 4.7. RNA Extraction and Quantitative RT-PCR

RNA was extracted and quantitative reverse transcription polymerase chain reaction (RT-PCR) was carried out as described previously [6].

### 4.8. Statistical Analysis

Data were analyzed using Graph-Pad InStat version 3.00 and Graph-Pad Prism version 5.02 softwares (GraphPad Software, San Diego, CA, USA). The results are presented as mean + standard error of the mean (SEM). One-way ANOVA and repeated measures two-way ANOVA with Bonferroni’s multiple comparison test were used in the statistical analyses as indicated in figure legends.

## Data Availability

All data is included in the manuscript.
